# Optic Nerve Sheath Diameter Assessment in Patients with Intracranial Pressure Monitoring

**DOI:** 10.7759/cureus.3546

**Published:** 2018-11-05

**Authors:** Tapan Kavi, Anuj Gupta, Krystal Hunter, Craig Schreiber, Hamza Shaikh, Alan R Turtz

**Affiliations:** 1 Neurology, Cooper University Hospital, Camden, USA; 2 Radiology, Cooper University Hospital, Camden, USA; 3 Biostatistics, Cooper Univerity Hospital, Camden, USA; 4 Neurosurgery, Cooper University Hospital, Camden, USA

**Keywords:** neuromonitoring, noninvasive, neurocritical care, optic nerve sheath diameter, eyeball transverse diameter, intracranial pressure (icp)

## Abstract

Background

Optic nerve sheath diameter (ONSD) has been found to have good correlation with intracranial pressure (ICP) measurements. Here, we aim to determine if the correlation between ONSD and ICP persists throughout the acute phase of neurologic injury through the evaluation of patients with ICP monitoring. We also aim to determine if the ONSD assessments at different depths (3, 6, or 9 mm) and a ratio of the ONSD and eyeball transverse diameter (ETD) are better correlated with ICP than the well-studied ONSD assessment at 3 mm beyond the globe.

Methods

This retrospective study included 68 patients more than 18 years of age with ICP monitors with both traumatic and spontaneous intracranial injuries. Head computed tomography (CT) scans were reviewed by a radiology resident for assessment of the ETD and ONSD at depths of 3, 6 and 9 mm beyond the globe, and the readings were confirmed by a neuroradiologist. The mean ICP recordings two hours before and after a CT scan were used for assessing the correlation.

Results

We found that ONSD expansions during the acute phase of neurologic injury were seen even without ICP elevations. This lack of correlation persisted even when different depths of the ONSD assessment or ONSD/ETD ratios were studied.

Conclusion

This study suggests that ONSD assessment throughout the acute phase may not be a reliable method to monitor ICP. ONSD expansion can persist even after ICP control, and this may be the reason for ONSD expansions seen in our study even with normal ICPs. Further larger size studies are needed to confirm these findings.

## Introduction

Optic nerve sheath diameter (ONSD) assessment has been shown in multiple studies to have good correlation with intracranial pressure (ICP) [1-6]. It is one of the few modalities for non-invasive assessment of ICP. The optic nerve is surrounded by cerebrospinal fluid and ensheathed by all three meningeal layers (dura, arachnoid, and pia mater), and thus intracranial pressure changes can be reflected through changes in ONSD. Non-invasive ICP assessment offers advantages over invasive means, such as an external ventricular drain or intraparenchymal pressure monitor, by reducing risks, such as hemorrhage and infection.

Both sonographic and radiologic assessments of ONSD have been studied. Multiple studies which include case reports, case series, retrospective studies, prospective studies, and meta-analysis have demonstrated the association of ONSD with ICP [1-7]. There have been very few studies showing a lack of this association. Recent reports have looked at assessing ONSD at a depth of greater than 3 millimeters (mm) beyond the globe and assessing a ratio of ONSD to eyeball transverse diameter (ETD) to account for differences in the size of the eyeball in patients [6, 8].

Firstly, we aim to determine if the association of ONSD and ICP persists throughout the acute phase of the neurologic injury through the evaluation of patients with ICP monitoring. Secondly, we aim to determine if the ONSD assessments at different depths (3, 6, or 9 mm) and the ratio of ONSD/ETD are better correlated with ICP than the well-studied ONSD assessment at 3 mm beyond the globe.

## Materials and methods

Protocol approval

This retrospective chart review was performed after approval from the Cooper University Hospital Institutional Review Board.

Study design

Patients with an ICP monitoring device placement (external ventricular drain or intraparenchymal pressure monitor) for traumatic or non-traumatic brain injury were identified during the five year study period, of which 73 patients were randomly selected based on a computer algorithm. Five patients were excluded because of incomplete data on chart review.  

Inclusion/exclusion criteria

Patients with ICP monitoring device placement, more than 18 years of age, and admitted between January 2012 and December 2016 were included in the study. Patients with trauma to the orbit or known ophthalmological disorders were excluded from the study.

Data collection

**Figure 1:**
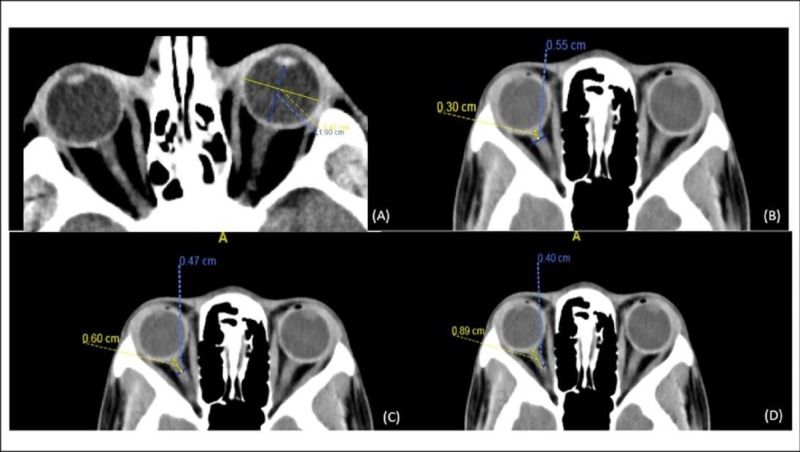
Optic Nerve Sheath Diameter (ONSD) and Eyeball Transverse Diameter (ETD) Measurement A) Eyeball transverse diameter measurement technique on computed tomography: Yellow line for ETD measurement is the largest line through the eyeball drawn perpendicular to the axis of the ONSD (blue line); B) shows a measurement of ONSD at 3 mm distance, (C) shows ONSD at 6 mm distance, and (D) shows the ONSD at 9 mm distance.

Clinical variables included age, Glasgow Coma Score (GCS) on admission, mortality, pupillary reactivity, and mean of intracranial pressure readings two hours before and after the CT scan were selected for collection of radiological variables. The radiological variables included ONSD at 3, 6, and 9 mm beyond the globe, ETD, and pattern of injury. This data collection was first performed by a radiology resident and then all measurements were confirmed by a board-certified neuroradiologist. The transverse diameter of the eyeball was chosen because the ONSD measurement was also in transverse section. Toshiba 320 and 64 slice scanners were used with 5 mm thickness and slice intervals. An example of the measurement technique is shown in Figure 1.

Statistical analysis

Statistical tests used were the Spearman rho correlation and Mann Whitney U Tests. The Spearman rho correlation was used to examine the relationship between the mean ICP and left and right ONSD measures and ONSD/EDT measures. The Mann Whitney U tests were used to compare the ranks of median ICP at different depths between those who had ONSDs less than or equal to 6 mm vs greater than 6 mm.

## Results

As can be seen in Table 1, subarachnoid hemorrhage, intraventricular hemorrhage, intracerebral hemorrhage, and subdural hemorrhages were the most common radiological findings. This represents a good mixture of both spontaneous and traumatic intracranial injuries. With regards to the severity of the injury, patients had moderate to severe injury based on a mean GCS of 8 at presentation and a mortality rate of 37% in the group. Moreover, the requirement for intracranial pressure monitor placement also reflected a high severity of the intracranial injury. However, the median ICP recording for two hours before and after the neuroimaging studies was not elevated (median ICP of 9 mm Hg), likely because the ICPs were stabilized for patients to be able to travel for the study.

**Table 1:** Baseline Characteristics GCS: Glasgow Coma Score; ICP: intracranial pressure; IQR: interquartile range

Number of patients	68
Mean age	50 years
Mean GCS on presentation	8
Mortality	37%
Pupillary reactivity on initial exam	76%
Intraventricular hemorrhage	47%
Subarachnoid hemorrhage	54%
Subdural hemorrhage	47%
Intracerebral hemorrhage	54%
Epidural hemorrhage	5%
Median ICP (IQR)	9 mm Hg (5 - 13 mm Hg)

We studied ONSD not just at a depth of 3 mm beyond the globe but also at 6 and 9 mm because of previous reports of an association between ICP and ONSD at depths beyond 3 mm. As seen in Table 2, ONSD on both right and left sides tended to decrease as the depth increased, and correspondingly, the ratio of ONSD/ETD also decreased. There was good symmetry in values for both ONSD and ETD between the right and left sides. The mean values for both ONSD and ONSD/ETD were less than previously reported values associated with elevated ICPs of 0.6 cm and 0.3, respectively.

**Table 2:** Optic Nerve Sheath Diameter (ONSD) and Optic Nerve Sheath Diamater/Eyeball Transverse Diameter (ONSD/ETD) Measurements IQR: interquartile range; L:  left; R; right

	Median measurement (IQR)		Median measurement (IQR)
R ONSD at 3 mm in cm	0.57 (0.53 - 0.63)	L ONSD at 3 mm in cm	0.55 (0.52 - 0.61)
R ONSD at 6 mm in cm	0.48 (0.44 - 0.52)	L ONSD at 6 mm in cm	0.49 (0.46 - 0.52)
R ONSD at 9 mm in cm	0.43 (0.38 - 0.48)	L ONSD at 9 mm in cm	0.43 (0.40 - 0.46)
Average of 3, 6, and 9 mm R ONSD in cm	0.50	Average of 3, 6, and 9 mm L ONSD in cm	0.50
R ETD	2.26 (2.20 - 2.32)	L ETD	2.25 (2.20 - 2.30)
R ONSD/ETD at 3 mm	0.25 (0.23 - 0.28)	L ONSD/ETD at 3 mm	0.25 (0.23 - 0.28)
R ONSD/ETD at 6 mm	0.21 (0.19 - 0.24)	L ONSD/ETD at 6 mm	0.22 (0.20 - 0.23)
R ONSD/ETD at 9 mm	0.19 (0.17 - 0.21)	L ONSD/ETD at 9 mm	0.19 (0.17 - 0.20)
Average of 3, 6, and 9 mm R ONSD/ETD	0.23	Average of 3, 6, and 9 mm L ONSD/ETD	0.22

**Figure 2:**
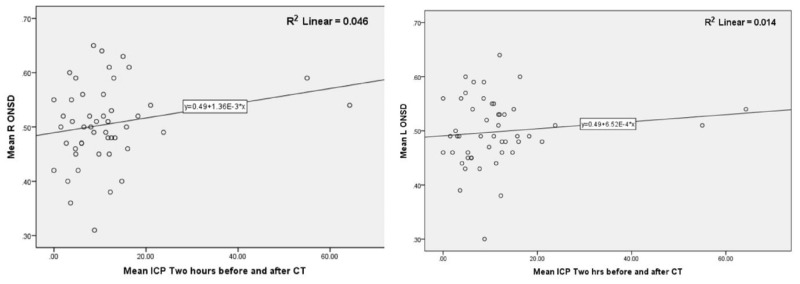
ONSD and ICP Correlation with a Scatter Diagram Scatter diagram showing lack of correlation between ICP and mean ONSD. The mean ICP two hours before and after CT head is plotted on x-axis and mean ONSD in cm on y-axis. CT: computed tomography; ICP: intracranial pressure; L: left; ONSD: optic nerve sheath diameter; R: right; R^2^: R-squared

We studied the relationship between ONSD, ONSD/ETD, and ICP using Spearman's rho correlation. As seen in Table 3, we did not find any significant correlation between ICP and ONSD or ONSD/ETD. The lack of correlation was seen across all depths studied. Figure 2 shows the lack of correlation between ICP and mean ONSD measurements.

**Table 3:** Correlation Among Intracranial Pressure (ICP), Bilateral ONSD, and Bilateral ONSD/ETD ETD: eyeball transverse diameter; ICP: intracranial pressure; L: left; ONSD: optic nerve sheath diameter; R: right

		R ONSD at 3 mm	R ONSD at 6 mm	R ONSD at 9 mm	R ONSD/ETD at 3 mm	R ONSD/ETD at 6 mm	R ONSD/ETD at 9 mm
Mean ICP	Correlation Coefficient	0.201	0.288	0.257	0.173	0.253	0.234
	Sig. (2-tailed)	0.172	0.064	0.120	0.238	0.106	0.158
	N	48	42	38	48	42	38
		L ONSD at 3 mm	L ONSD at 6 mm	L ONSD at 9 mm	L ONSD/ETD at 3 mm	L ONSD/ETD at 6 mm	L ONSD/ETD at 9 mm
Mean ICP	Correlation Coefficient	0.137	0.226	0.095	0.108	0.193	0.078
	Sig. (2-tailed)	0.349	0.145	0.553	0.460	0.215	0.628
	N	49	43	41	49	43	41

We also studied changes in ICP with previously reported thresholds associated with elevated ICP − 0.6 cm for ONSD and 0.3 for ONSD/ETD. As can be seen in Table 4, there was no statistically significant increase in the median ICP between the cut-off of 0.6 cm for ONSD at any depth. We did not have enough data points to study the change in ICP for the ONSD cut-off of 0.6 cm at 9 mm depth on the right side and 3 mm and 9 mm depth on the left side. There was a statistically significant increase in ICP for ONSD/ETD cut-off of 0.3 on the right side (9.02 mm Hg to 12.5 mm Hg); however, no significant increase was seen for the left side. Also, although the ICP change was statistically significant, it was lower than the threshold for the diagnosis of elevated intracranial pressure (20 mm Hg). The ONSD/ETD ratio on the right side at 3 mm was seen to have a statistically significant increase when a midline shift of more than 5 mm was present using the Mann Whitney U test (P = 0.037). The ratio increased from 0.248 to 0.278 when a midline shift of 5 mm or more was present; however, it was still lower than the previously identified threshold for elevated ICP (0.3). The presence of unilateral lesions and midline shift could be one explanation for the statistically significant increase in ICP for ONSD/ETD cut-off on the right side but not the left side. Again, we did not have enough data points to study the change in ICP for the ONSD/ETD cut-off of 0.3 at depths of 6 and 9 mm on either side.

**Table 4:** Median ICP for ONSD and ONSD/ETD Cut-off ETD: eyeball transverse diameter; ICP: intracranial pressure; L: left; ONSD: optic nerve sheath diameter; R: right

	Median ICP for depth of 3 mm on R side	Median ICP for depth of 6 mm on R side	Median ICP for depth of 9 mm on R side	Median ICP for depth of 3 mm on L side	Median ICP for depth of 6 mm on L side	Median ICP for depth of 9 mm on L side
ONSD ≤​​​​​​​ 0.6 cm	9.02	10.75	10.25	9.02	8.80	9.75
ONSD > 0.6 cm	10.58	8.60	-	10.75	-	-
P value with Mann Whitney U test	0.988	0.226	-	0.689	-	-
	Median ICP for depth of 3 mm on R side	Median ICP for depth of 6 mm on R side	Median ICP for depth of 9 mm on R side	Median ICP for depth of 3 mm on L side	Median ICP for depth of 6 mm on L side	Median ICP for depth of 9 mm on L side
ONSD/ETD ≤​​​​​​​ 0.3	9.02	10.25	10.25	9.23	8.80	9.75
ONSD/ETD > 0.3	12.5	-	-	9.25	-	-
P value with Mann Whitney U test	0.026	-	-	0.783	-	-

In summary, we did not find a statistically significant correlation between ICP and ONSD or ONSD/ETD. Previously reported thresholds of ONSD or ONSD/ETD for elevated ICP were not found to be associated with elevated ICP in our study, suggesting that expansion in ONSD is not specific for elevated ICP. 

The results of this study were presented at the Neurocritical Care Society (NCS) 16th Annual Meeting in 2018. (P41: Teklemariam E, Kavi T, Prasanth R, Gupta A, Hunter K, Schreiber C, Shaikh H, Turtz A: Optic nerve sheath expansion is not specific for elevated intracranial pressure. Neurocritical Care Society (NCS) 16th Annual meeting, Boca Raton, FL, Sept. 25-28, 2018)

## Discussion

ONSD measurements via ultrasound or radiographic imaging have been shown in many previous studies to be predictive of elevated ICP [1-7]. ONSD measurement of 5 mm or higher at a depth of 3 mm beyond the globe has been shown to have good correlation with ICP elevation beyond 20 mm Hg [1-4]. Noninvasive measuring of intracranial pressure is advantageous as it allows physicians to assess the intracranial environment without the risks of infection and hemorrhage associated with conventional invasive techniques. Another theoretical advantage with non-invasive modalities, like ultrasound, is the ability to evaluate a patient’s ICP quickly at the bedside. This allows the more judicious use of invasive modalities like ventriculostomy. Although most previous studies have looked at assessing ONSD through ultrasound, studies have shown a good correlation of ultrasound with both CT and MRI measured ONSD [4-5].

Measuring the optic nerve sheath diameter at 3 mm has recently been challenged as the ideal distance for measuring. Some literature advocates for measurements of diameter at 8 to 10 mm beyond the globe for a more accurate assessment of the intracranial pressure. The theory for measuring an ONSD farther from the globe is that the scleral and dural attachments may limit ONSD expansion with increasing elevations in ICP. A potential downfall of using ONSD measurements only is the difference in size of the eyeball from person to person. The ratio of ONSD/ETD is an attempt to account for the normal variation in globe phenotype, and it may be more sensitive and specific for ICP assessment [6, 8]. This ratio was included in our study to evaluate its correlation with ICP measurements in comparison to standard ONSD measurements.

The major finding from this study was the lack of correlation between ONSD and ICP when followed during the acute phase of neurologic injury. Although this finding appears to show contrasting results from previously reported findings of a good correlation between ONSD and ICP, there are important differences. Our study included ICP measurements at varying stages during the patient's hospitalization. Some of them represented a more acute scenario, while others were later during the course. Previous studies did not have ONSD assessments necessarily after ICP control and may reflect a more immediate phase of neurologic injury. It is possible that ONSD has better correlation with ICP in the initial stages, and ONSD changes may persist even after control of ICP has been obtained. The retrospective nature of this study makes it difficult to standardize when the ONSD assessment is performed in relation to the patient course. However, this also lends a possible insight into the trend of ONSD changes with ICP control. The other major difference was that this study did not have many ICP measurements above 20 mm Hg, which is commonly used for diagnosis of intracranial hypertension. Therefore, the results of this study relate more to the specificity of ONSD for diagnosis of elevated ICP and not sensitivity, which has been studied more in previous reports. Thus, this study does not refute the findings from the previous studies but supplements them by adding data for the specificity of ONSD in relation to ICP and trends in ONSD changes after ICP control.

The small sample size in this study is also a limitation. Although a statistically significant change in ICP was seen on the right side only for ONSD/ETD cut-offs, it was still below the threshold for diagnosis and treatment of elevated intracranial pressure. To our knowledge, this is the first study showing a unilateral change in ONSD with ICP. This change could be related to the rise in ONSD/ETD seen on the right side only with midline shift in our study; however, given the small sample size, these findings need to be confirmed by a larger scale study. A prospective study that standardizes the timing and acuity of ICP measurement with subsequent radiologic studies would provide much stronger data for non-invasive ICP monitoring using ONSD or ONSD/ETD.

## Conclusions

This study lends a possible insight into the trend of ONSD changes with ICP control. ONSD expansion can persist even after ICP control, and this may be the reason for ONSD expansions seen in our study even with normal ICPs. ONSD assessment throughout the acute phase may not be a reliable method to monitor intracranial pressures and further larger size studies are needed to confirm these findings.
